# Supplementary material for: Host chitinase 3-like-1 is a universal therapeutic target for SARS-CoV-2 viral variants in COVID-19
Source: eLife. 2022 Jun 23;11:e78273. doi: 10.7554/eLife.78273 (PMC9273216; doi:10.7554/eLife.78273)
Supplement: Figure 4—source data 1. [file elife-78273-fig4-data1.pdf]

Uncut full gel photo for Western blots used in Figure 4.

Figure 4A

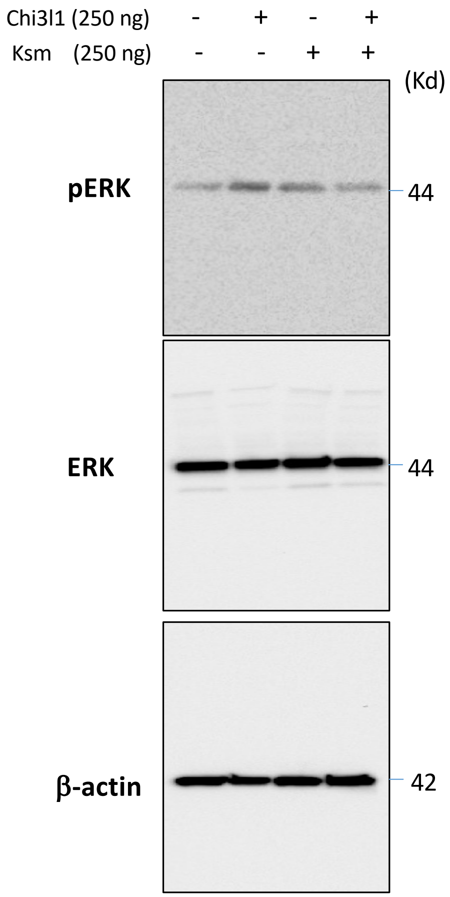

Figure 4B

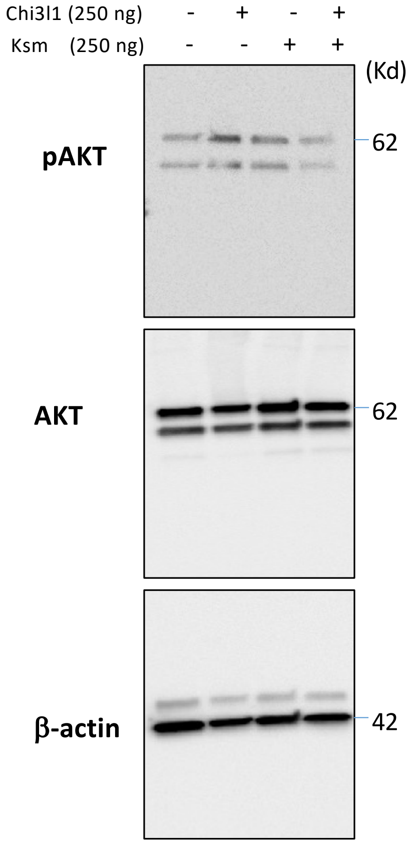

Densitometry data

TErk 20,831,109 26,395,746 26,838,669 22,284,795
TAKT 38,741,934 41,730,528 50,292,858 37,472,904
PERK 335,790 745,836 494,936 366,964
BACTIN 27,213,459 21,010,665
PAKT 208,736 836,480 551,328 303,968
B-ACTIN 26,608,647 28,609,347 3,676,140 9,860,838
