# Supplementary material for: Host chitinase 3-like-1 is a universal therapeutic target for SARS-CoV-2 viral variants in COVID-19
Source: eLife. 2022 Jun 23;11:e78273. doi: 10.7554/eLife.78273 (PMC9273216; doi:10.7554/eLife.78273)

Fig.6. Immunocytotochemical evaluation of delta pseudovirus infection of Calu-3 cells. Box in each panel indicates the selected representative area used in figure 6.

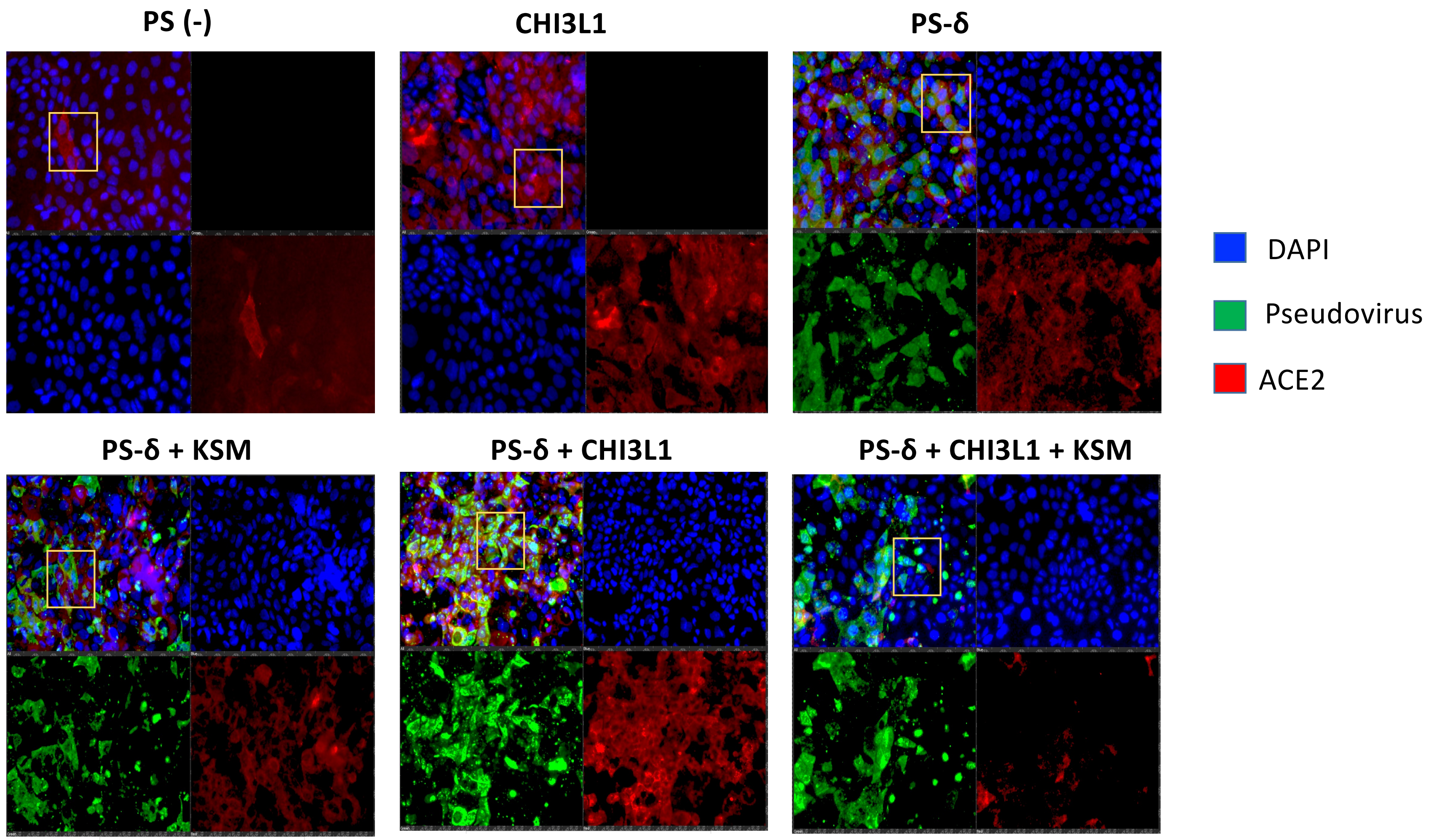

Supplement: Figure 6—source data 1. [file elife-78273-fig6-data1.pdf]
