# Supplementary material for: Host chitinase 3-like-1 is a universal therapeutic target for SARS-CoV-2 viral variants in COVID-19
Source: eLife. 2022 Jun 23;11:e78273. doi: 10.7554/eLife.78273 (PMC9273216; doi:10.7554/eLife.78273)
Supplement: Supplementary file 1. [file elife-78273-supp1.docx]

**Supplementary File 1. Pseudoviruses containing S protein mutations of COVID variants used in this study**

| **Variant Name** | **Mutations in S protein** | **Pseudovirus Source (Cat #)** |
| --- | --- | --- |
| Alpha (B.1.1.7 and Q lineages) | Deletions of H69, V70, and Y144; N501Y, A570D, D614G, P681H T716I, S982A, D1118H | BPS BIOSCIENCE Inc. Cat#78112-1 |
| Beta (B.1.351 and descendent lineages) | L18F, D80A, D215G, R246I, K417N, E484K, N501Y, D614G A701V | BPS BIOSCIENCE Inc,  Cat #78142-1 |
| Gamma (P.1 and descendent lineages) | L18F, T20N, P26S, D138Y, R190S K417T, E484K, N501Y, D614G H655Y,T1027I | BPS BIOSCIENCE Inc.  Cart #78144-1 |
| Delta (B.1.617.2 and AY lineages) | T19R, G142D, 156/157 Deletion, R158G, L452R,T478K, D614G, P681R, D950N | BPS Bioscience Inc.  Cat# 78216-1 |
| Omicron (B.1.1.529 and BA lineages) | S371L, G339D, S375F, S373P, K417N, N440K, G446S, S477N, T478K, D614G, E484A, Q498R, H505Y, N501Y, Q493R | eEnzyme.com  SCV2-PsV-Omicron |
